# Book Reviews

**Published:** 1809-06

**Authors:** 


### CRITICAL ANALYSIS
### OF THE
### RECENT PUBLICATIONS
### ON THE
### DIFFERENT BRANCHES OF PHYSIC, SURGERY, AND MEDICAL PHILOSOPHY.

---

*Observations on Madness and Melancholy, including Practical Remarks on those Diseases; together with Cases, and an Account of the morbid Appearances on Dissection.* By JOHN HASLAM, late of Pembroke Hall, Cambridge, Member of the Royal College of Surgeons, and Apothecary to Bethlem Hospital. Second Edition, considerably enlarged. London, 1809, pp. 345.

IN all Dissertations on Madness, one great difficulty is to ascertain the disease, that is, to offer a satisfactory definition. The etymologies proposed by our author are very judicious; but if madness means only rage, and sorrow nothing but soreness, we are not to rest with a mere knowledge of the opinions of our ancestors, or of the imperfect manner in which the poverty of their language obliged them to clothe their ideas. After remarking on the present copiousness of our language, which enables us to vary the terms, Mr. Haslam concludes with observing, " how much more advantage would be obtained if the circumstances could be precisely defined, under which it is justifiable to deprive a human being of his liberty." Previously to such an undertaking, it is very justly observed, that we should ascertain what the state of the human mind is when sound; "and (it is added) in a legal point of view, it is most important that the medical practitioner should be enabled to establish the state of the patient's case, as a departure from that which is reason."

Next to this we have an account of the metaphysicians' opinions concerning madness. After all that has been said, they amount, we think, to nothing more than a defect of judgment; for though the cobler, in fancying himself an emperor, may exhibit an elevated flight of imagination, yet, as our author observes, "it is a great defect in his judgment to deem himself what he is not; and it is certainly an equal lapse of his recollection to forget what he really is."

As to the flight of his imagination, we are at a loss completely to comprehend the meaning of the expression in this place. When a striking event impresses the mind with images somewhat remote, yet readily excited by that event; and when the impression of these images is related in language appropriate, and at the same time well directed to the impression, we shall then say that the imagination is bold in proportion as the images are grand; well directed in proportion as they bear upon the event; and well described, though flighty in proportion to the abruptness with which they intrude themselves on the person, or he communicates them to others. In all this, judgment is absolutely necessary, nay implied, by the very statement of the question. For if the images excited are no way connected with the event, *Romani tollent patres, equitesque cachinnum.* If they are ill described, the hearer will yawn; if they are so abrupt that he cannot follow the association, he will stare. If this be admitted, the whole reverts into judgment. The cobler's memory cannot be so defective as not to inform him that the insignia of royalty are not the awl and the strap, but his judgment may be so deficient as to prevent him from properly combining events immediately passed, or at that time actually before him.

Mr. Dugald Stewart, it is remarked, "by conception, means that power of the mind which enables it to form a notion of an absent object of perception, or of a sensation which it has formerly felt." "This definition, adds our author, means merely memory." If it means nothing more than memory, it is absurd to give it any other name. It however extends a little further, because a clear notion of the object is implied; even unless it means more than this, it only extends to a *correct* memory. But if Professor Stewart means by the *absent object*, one of which the hearer knows no more than by the relation of others, he is then perfectly correct, because it will then be the *catching* from another a series of ideas, which that other gained by actual observation, perhaps through the medium of a different sense, of all his senses, or by long long application. Thus an able pugilist, who has seen a battle, will retain every part of its history; and if he relates it well to an adept, the latter will *catch* the intention of the relator in every particular; that is, have a clear *conception* of the whole. Or a lawyer, who hears the tale of his client, even if ill related, will *catch* his meaning, and gain a clear *conception* of the case. In both these instances judgment is required.

A digression, by no means uninteresting, follows, on the opinions concerning demoniacal possessions; after this, the general division of Mania and Melancholy is introduced. The first, according to Dr. Ferriar, consisting of " false impressions, and consequently confusion of ideas." The latter is said, by the same writer, " to consist in intensity of idea, which is contrary to false impressions." We are by no means satisfied with these definitions; and without in the least wishing to undervalue Mr. Haslam, we confess that his Remarks are, to us, not sufficiently perspicuous. We shall reserve our own attempt at definition till we have considered Mr. H's account of symptoms. This Chapter concludes with the fellowing judicious remarks.

" As the practitioner's own mind must be the criterion, by which he infers the insanity of any other person; and when we consider the various, and frequently opposite, opinions of these intellectual arbitrators; the reader will be aware that I have not abstained from giving a definition of madness without some reason. There is indeed a double difficulty: the definition ought to comprise the aberrations of the lunatic, and fix the standard for the practitioner.

" But it may be assumed that sound mind and insanity stand in the same predicament, and are opposed to each other in the same manner, as right to wrong, and as truth to the lie. In a general view no mistake can arise, and where particular instances create embarrassment, those most conversant with such persons will be best able to determine.

" The terms sound mind and insanity are sufficiently plain. If to an ordinary observer, a person were to talk in an incoherent manner, he would think him mad; if his conduct were regular, and his observations pertinent, he would pronounce him in his senses: the two opposite states, well marked, are well understood; but there are many different shades, which are not so likely to strike the common examiner."

The next Chapter is on the symptoms of the disease. On this subject we are much pleased to see our author, as indeed we might expect, object to those numerous and useless nosological, or, as they might be better termed, philoepic distinctions, which can serve no useful purpose. As in people of sound mind it is very justly remarked, so in madmen it may with equal justice be said, that each individual has a certain character by which he may be distinguished from the rest of the species; and this variety of character is not less universal than the variety of countenance. To mark mark all these little changes in madmen, would be not less useless than to divide the human character into orders, genera, species, and varieties. The following are offered by Mr. H. as the early symptoms.

"On the approach of mania, they first become uneasy,* are incapable of confining their attention, and neglect any employment to which they have been accustomed; they get but little sleep, they are loquacious, and disposed to harangue, and decide promptly, and positively upon every subject that may be started. Soon after, they are divested of all restraint in the declaration of their opinions of those, with whom they are acquainted. Their friendships are expressed with fervency and extravagance; their enmities with intolerance and disgust. They now become impatient of contradiction, and scorn reproof. For supposed injuries, they are inclined to quarel and fight with those about them. They have all the appearance of persons inebriated, and those who are unacquainted with the symptoms of approaching mania, generally suppose them to be in a state of intoxication. At length suspicion creeps in upon the mind, they are aware of plots, which had never been contrived, and detect motives that were never entertained. At last the succession of ideas is too rapid to be examined; the mind becomes crowded with thoughts, and confusion ensues.

"Those under the influence of the depressing passions, will exhibit a different train of symptoms. The countenance wears an anxious and gloomy aspect, and they are little disposed to speak. They retire from the company of those with whom they had formerly associated, seclude themselves in obscure places, or lie in bed the greatest part of their time. Frequently they will keep their eyes fixed to some object for hours together, or continue them an unequal time "bent on vacuity." They next become fearful, and conceive a thousand fancies: often recur to some immoral act which they have committed, or imagine themselves guilty of crimes which they never perpetrated: believe that God has abandoned them, and, with trembling, await his punishment. Frequently they become desperate, and endeavour by their own hands to terminate an existence, which appears to be an afflicting and hateful incumbrance."

Some judicious remarks follow, on what are called lucid intervals, and particularly on the uncertainty of them, to those who are not much accustomed to madmen. The memory, we are next informed

* "In many instances, although it is far from being general, pain of the head, and throbbing of its arteries precede an attack of insanity; sometimes giddiness is complained of as a precursory symptom. Those who have been several times disordered, are now and then sensible of the approaching return of their malady. Some have stated, a sense of working in the head, and also in the intestines, as if they were in a state of fermentation. Others observe that they do not seem to possess their natural feelings, but they all agree that they feel confused from the sudden and rapid intrusion of unconnected thoughts."

informed, is very soon deficient in madness, and some remarks are offered on the cause of this. To us they appear to be all reducible to the following. That in all persons the memory of an event is lively, permanent, and correct, in proportion as the impression is stronger, and as the mind is accustomed to order. That the recollection must depend in some measure on the same causes, but be much influenced by the mode in which subsequent impressions have become stronger, and most of all, in proportion as the mind is so far at ease, respecting all other impressions, as to call up the images of a past event by the slightest association. To illustrate our meaning, we will suppose a person, at a middle or advanced age, of a disposition to be pleased, during whose early years many events occurred, which, from their novelty at that age, were both striking and agreeable to him; he will feel a pleasure in dwelling on such events; and many occurrences, several of them apparently remote, will revive the recollection of them, which, in some cases, become so habitual as to be called up whenever the mind is no otherways occupied. It is on this account that we so often see blind people with a smile, which to many appears somewhat vacant. When no surrounding objects engage their attention, they naturally call up those images which are most agreeable to them; but when engaged in conversation, their countenance varies like other peoples'

To apply this to our present subject. When the mind is at ease, the recollection of every event will be lively in proportion as the impression it produced at the time was strong; and it will recur with a frequency proportionate to the gratification it affords. This gratification will depend not entirely upon the pleasure which was felt at the time, but upon the character of the person, or the general frame of his mind. If it be naturally cheerful, the recollection of cheerful events will be so agreeable, that the slightest resemblances will revive them, and in his hours of solitude he will amuse himself by retracing them. Even on gloomy events, if unattended with depravity, he will learn to reflect with sentiments not entirely painful. The remembrance of past dangers which he has escaped, or sufferings he has submitted to, serve to heighten his present enjoyment. But all this requires a security, or at least a complacency, arising from a supposed security. To a gloomy mind, or to one whose prospects are gloomy, whatever has an air of peculiar cheerfulness, forms only a contrast to its own state, which heightens its misery; and though it can have no satisfaction in recollecting cheerful events, yet however accordant gloomy ones may be with its present state, even to dwell on them is painful. The memory, therefore, is only perfect in those events which are useful or necessary. Now, usefulness and necessity, like every thing else, are comparative. To a mind under very strong impressions from any cause, all the common events of life lose their importance; nothing engages it but the present scene; and it can dwell on nothing else. But to the madman, during his paroxysms, the the scene is not what is really passing before him; his ideal impressions are too strong to permit the recollection of any other, or too strong to permit his entertaining such recollections if they do occur. This may be further illustrated by what has been said of the similarity between madness and dreaming.

When sleep is perfectly sound, no dreaming attends it; or if any, the impression is so slight, as rarely to be recollected. When sleep is less perfect, it is only a higher degree of that state which we call reverie; that is, the senses are all so torpid, that we are regardless of every thing about us, and the suggestions of our own fancy pass with us for realities. The only difference between these two states is, that in dreaming the organs of sense are more torpid, and the means of exciting one set are for a time lost, the eye-lids being closed. In the reverie, therefore, the mind may recover its true impressions from surrounding objects by any sudden appearance opposed to the sight, as well as by any slight stimulus upon the other senses. In sleep it is necessary to stimulate the other senses, and that in proportion to the soundness of the sleep. —Let us now suppose a reverie, induced by a cause likely to be more permanent, arising not from an event suddenly occurring, which for a time occupies the whole mind; not from a constitutional peculiarity, which is apt to be more indifferent to common objects; nor from that oblivion of the senses, which after a time follows their diurnal exertions in most of the animals we are acquainted with; but from some local derangement in those organs, whose regular actions are necessary to convey to the mind impressions conformable to the object presented to the senses. In this case, whatever ideas occur to the mind may pass for realities, and even the most powerful impressions we can make on the senses, may not be sufficient to excite in the mind corresponding images and their regular successions. Such an event as this may arise from one of the causes above stated; that is, if the mind be suddenly and powerfully affected by any event, a more than ordinary action may be produced on those organs which convey the proper impression by means of the senses. In this case it will be in vain to expect that the chain or succession which usually follows from the application of an object to the senses should be complete. The mind is incapable of receiving an impression from new images whilst the event keeps entire possession of it; and this unnatural state may continue so long as to injure the organ thus powerfully impressed. In which case, though the immediate effect of the impression may cease, yet those corresponding actions by which the senses convey to the mind the impressions they feel, will be lost.

All this time the senses may be perfect in themselves. With respect to most of them, it will be impossible to ascertain such a fact; but in one we often see it realized. In cases of spina bifida, or more properly, of spine distorted by disease, the lower extremities may be paralyzed; and though, under these circumstances, the muscles will be as susceptible of stimuli as before, yet the mind is totally insensible of every application made to them.

To apply all this to madness. From such a state as we have supposed, it will be in vain to expect correct memory, or judgment; the mind is too much occupied to entertain a regular recollection of past events, or to combine those objects which are presented to it; some real or imaginary event is always present.—If the recollection is agreeable, it will be perpetually called up, and entertained as it were by force; if painful, it will obtrude itself in spite of the will, and its impression will be too powerful to admit of any other, excepting as a momentary effect. The only remedy therefore that remains for the unhappy object, is to invent if possible, some means of disengaging himself from the effect of impressions which are always obtruding themselves. On this subject we shall quote a passage from our Author, which shows, in a lively manner, the similarity between reverie and madness.

"Many of those who are violently disordered will continue particular actions for a considerable time: some are heard to gingle the chain, with which they are confined, for hours without intermission; others, who are secured in an erect posture, will beat the ground with their feet the greatest part of the day. Upon enquiry of such patients, after they have recovered, they have assured me that these actions afforded them considerable relief. We often surprize persons who are supposed free from any mental derangement, in many strange and ridiculous movements, particularly if their minds be intently occupied:*—this does not appear to be so much the effect of habit, as of a particular state of the mind."

That Johnson was, in many respects, "just not mad," seems pretty generally admitted; and that his madness was of a gloomy cast is as well known. His biographer informs us, he was often talking to himself, and that on these occasions there was reason to believe

* "The late Dr. Johnson was remarkably distinguished by certain peculiarities of action when his mind was deeply engaged. Sir Joshua Reynolds was of opinion "that it proceeded from a habit he had indulged himself in; of accompanying his thoughts with certain untoward actions." "One instance of his absence and peculiarity, as it is characteristic of the man, may be worth relating. When he and I took a journey into the West, we visited the late Mr. Banks, of Dorsetshire; the conversation turning upon pictures, which Johnson could not well see, he retired to a corner of the room, stretching out his right leg as far as he could reach before him, then bringing up his left leg, and stretching his right still further on. The old gentleman observing him, went up to him, and in a very courteous manner assured him, that though it was not a new house, the flooring was perfectly safe. The Doctor started from his reverie like a person *waked out of his sleep*, but spoke not a word."—*Boswell's Life of Dr. Johnson, vol* i. p. 176. In the same work, other of his tricks are recorded, as talking to himself, measuring his steps in a mysterious manner, half whistling, clucking like a hen, rubbing his left knee, &c. Many sensible persons, with whom I am now acquainted, when particularly thoughtful, discover strange bodily motions, of which they are by no means conscious at the same time."

believe that he was endeavouring to divert from his mind some impression which was painful to him. This is not uncommon with many men of strong feelings, or with such as are easily diverted from the impression of events passing before them: such we mean as are usually called *absent*. Thus Mr. Toby Shandy, when any thing occurred which was not agreeable to him, instead of complaining, whistled *Lillebulero*. When those subjects took place, which were most agreeable to him, he could think on nothing else; and on some occasions the impressions were so powerful that he could only view them in his mind's eye; and this complacency would be so grateful, that even the interruption of conversation, though on the topic itself, would be troublesome and intruding. Thus, when the Corporal suggested leaving town, and amusing themselves with counterscarps and the other insignia of seiges, the Captain was so entirely impressed with the subject, that he could not even admit the prattling of the proposer, because every thing, and more than he suggested, was anticipated in his own mind.

"By some," says our Author, "madness has been compared to dreaming."—

"There is, however, a circumstance, which to my knowledge, has not been noticed by those who have treated on this subject, and which appears to establish a marked distinction between madness and dreaming. In madness, the delusion which we experience is most frequently conveyed through the ear; in dreaming, the deception is commonly optical; we see much, and hear little; indeed dreaming, at least with myself, seems to be a species of pantomine, that does not require the aid of language to explain it. It is true, that some who have perfectly recovered from this disease, and who are persons of good understanding and liberal education, describe the state they were in, as resembling a dream: and when they have been told how long they were disorded, have been astonished that the time passed so rapidly away. But this only refers to that consciousness of delusion, which is admitted by the patient on his return to reason; in the same manner as the man awake, smiles at the incongruous images, and abrupt transitions of the preceding night. In neither condition, does the consciousness of delusion, establish any thing explanatory of the *state* of the mind."

If we understand our Author in this passage, he seems to admit there may be a similarity between dreaming and madness, but that this can only be ascertained in such as become convalescent, and even then, that we are ignorant of the state of mind in both cases. As to the different senses imposed upon in the two cases, this may be accounted for by what we mentioned before, when speaking of dreams, viz. the organ of sight is so closed, that the impression of images which arise in the mind, cannot be effaced by or contrasted with any real objects. This is not the case in madness. But even here the mind may be so much impressed with a particular idea as to be insensible to the impression of all external ternal objects of sight; or, as we say, that a person may be looking at nothing. In which case, the idea uppermost in the mind will pass for a reality, as in the following instances mentioned in a former part of Mr. Haslam's work.

"I have known many cases of patients who insisted that they had seen the devil. It might be urged, that in these instances, the perception was vitiated: but it must be observed there could be no perception of that, which was not present and existing at the time. Upon desiring these patients to describe what they had seen, they all represented him as a big, black man, with a long tail, and sharp talons, such as is seen pictured in books; a proof that the idea was revived in the mind from some former impressions. One of these patients however carried the matter a little further, as she solemnly declared, she heard him break the iron chain with which God had confined him, and saw him pass fleetly by her window, with a truss of straw upon his shoulder."

Here we see more of the pantomime than of any delusion of the ear. The fact seems, that in both cases the judgment is so defective, that whether sounds are heard, or objects seen, the mind cannot distinguish between realities and phantoms. In the reverie, and in dreaming, the impression only lasts till the senses are sufficiently roused, after which the judgment being sound, the illusion vanishes. If madness is unattended with organic lesion, the same may happen, though the illusion may continue longer. But if from a wrong action in the vessels of certain organs, the judgment cannot be restored, it will be of little consequence that the senses receive the true impression; the memory will still retain an event so impressive as "the big black man with the long tail." Nor is there any judgment to appeal to, in order to convince our patient that it was a delusion.

After enumerating the symptoms most to be relied on, Mr. H. refers to one which has been admitted without sufficient reserve. It is of much importance to remove this error; for which reason, we shall transcribe the passage, as producing an instance in which aur Author seems to us to consider reverie as an illustration at least of madness.

"Of the power which maniacs possess of resisting cold, the belief is general, and the histories which are on record truly wonderful: it is not my wish to disbelieve, nor my intention to dispute them; it is proper, however, to state that the patients in Bethlem Hospital possess no such exemption from the effects of severe cold. They are particularly subject to mortifications of the feet; and this fact is so well established from former accidents, that there is an express order of the house, that every patient under strict confinement, shall have his feet examined morning and evening in the cold weather by the keeper, and also have them constantly wrapped in flannel; and those who are permitted to go about, are always to be found as near the fire as they can get, during the winter season.

"From the great degree of insensibility which prevails in some states of madness, a degree of cold would scarcely be felt by such persons, which would create uneasiness in those of sound mind; but experience has shewn that they suffer equally from severity of weather. When the mind is particularly engaged on any subject, external circumstances affect us less than when unoccupied. Every one must recollect, that in following up a favourite pursuit, his fire has burned out, without his being sensible of the alteration of temperature; but when the performance has been finished, or he has become indifferent to it from fatigue, he then becomes sensible to cold, which he had not experienced before."

We are not surprised if madmen are more liable to mortification of the feet, because we shall presently see that there is in many an increased action going on in a very distant organ. But this only shows that the general frame of a madman is subject to the common laws of the animal economy, whilst from the diseased state of certain parts, the mind no longer receives the customary impressions of external objects conveyed by the senses. Thus, though in the reverie, or abstraction during a favorite study, the insensibility to cold, as well as to all external objects exists for a time, it ceases as the reverie ceases, or as the favorite study loses its *charm*, which will generally happen before the vessels of the toes have lost their power of recovering their proper actions; but this can only happen to the madman as he recovers his reason, or in other words, as he becomes less mad; and this period may be so distant, that in the mean time, the action of the vessels necessary to the support of the toes, may be irrecoverably lost. On the whole, therefore, we should define madness a reverie from which the patient cannot be roused, or a dream from the impression of which he cannot be relieved, or the illusion of which he is incapable of discovering, whilst his disease continues.

This leads to a consideration of the means of relief; and as it is obvious, that to relieve any disease, we must first learn the change produced in the organ with which that disease is connected, Mr. Haslam very judiciously, at this stage of his work, produces his cases, every one of which is so interesting, that after perusing the whole, we only regretted there were no more. As all such of our readers, who find it necessary to attend particularly to these subjects, will peruse the work, we shall only offer a few general remarks.

With only two exceptions out of 37 cases, the brain was found in a state which was likely to produce an alteration in its actions from those of health. In most, great congestion, in several inflammation, or the remains of it in the membranes in many a fluid of different descriptions, but generally limpid in the ventricles. The texture of the brain sometimes firm, at others soft, in one or two doughy, but in most natural. In one of the instances in which no appearances of disease could be discovered in the head, hæmiplegia occurred before death. The other which Mr. Haslam calls calls an additional example, appears to us less so. The patient, after being cured of dysentery, died suddenly. Though no extravasation of blood, or any other fluid was discovered in the brain, yet there was "a considerable determination of blood" to that organ.

In one case only, the brain appeared gangrenous. In one case the tunica arachnoidea was opake, and the veins seemed to *contain air.* In another, "the vessels of the pia mater were turgid, and seemed to contain air." One other we must particularly mark. "On the left posterior lobe of the cerebrum, there was a large quantity of a milky fluid between the tunica arachnoidea and pia mater, giving the appearance of a vesication, and in that place there was a depression or cavity formed in the convolution of the brain. *The convolutions were so strongly and distinctly marked, that they resembled the intestines of a child.*" Does this sanction the opinion of Gall on the nature of these convolutions?

Besides the one who suffered from dysentery, the history is given of another who died of that disease, and also of a female who died of phthisis. On the latter Mr. Haslam remarks,

"It is a common opinion, that phthisis pulmonalis is frequently suspended by the supervention of mania; medical books abound with such accounts, and some persons have supposed it difficult, if not impossible, for these diseases to co-exist. It is not my intention to dispute the accuracy of such relations, nor to question the power which mania may possess in arresting the progress of phthisis pulmonalis, but to state that the converse does not obtain; and, that whatever obligations may be due from phthisis to mania, the compliment has not been returned. From my own experience I can affirm, that insane persons are as liable to phthisis pulmonalis as others; that numbers of them die of that disease; and that I never saw any abatement of the maniacal symptoms through the progress of consumption."

We should hardly have thought this question deserved so serious a reply. In the early stage of phthisis, a violent diseased action set up in another part of the body, may be supposed to suspend the immediate progress of the first. But if the lungs have so far suffered, that tubercles are formed, we know that a very slight cause will bring them into suppuration; and it is well known how much dejection of mind will impede any curative process in these as well as any other diseased parts. There is, therefore, nothing more surprising in phthisis, in a maniac, than there would be in an ill-conditioned ulcer in the leg. Respecting many of those chronic diseases which seem to occupy if not the whole constitution, at least the whole nervous system, we have seen instances in which they have all ceased on the occurrence of confirmed mania. Probably they might have been only another form of the same disease.

We have next a chapter containing cases of insane children. They all seem to us cases of idiotcy in different degrees.

 Having

Having thus described insanity in its various stages, as well as the appearances on dissection, we are introduced to the causes of the disease. In these the author complains of the difficulties he has met with from the backwardness of the relations, to relate many particulars in the history of a maniac's complaint, and particularly such circumstances as may mark an hereditary pre-disposition. The greater part of this chapter is taken up in objections to the causes assigned by other writers. Hereditary disposition is, however, stated as well established, and proved by several well-ascertained facts. A few objections are started against the doctrine, but they are as nothing compared with the weight of evidence. The subject leads to several important inquiries, which we shall decline entering into, as the space allotted on the "Observations" themselves is much too limited. We wish some accurate reasoner, aware of all the weak points in Mr. Portal's paper, would undertake the subject, availing himself of the authorities produced by that author.

Having settled this question as far as the facts which have occurred to our author would permit, Mr. H. makes a few remarks on what are called the moral causes. These he traces principally to the errors of education.

Of the proximate, or as our author prefers calling it, the more immediate cause, he professes himself ignorant, referring all that he has to say on the subject to those diseased appearances in the brain, which he has before related. A doubt is indeed admitted, whether these appearances may be the cause or effect of madness; but as the same wrong actions arising from injury or common inflammation, will produce similar effects, he conceives there can be no necessity for calling in the aid of other causes. If we are to consider madness a disease of the mind, Mr. H. conceives that it would be in vain to offer remedies for what is supposed to be immaterial. We cannot see the least necessity for introducing the subject of immateriality, a word without a meaning, as our Author very properly remarks, and therefore a word on which we can form no proposition. But if during a disease in the brain from any cause, a habit has been acquired of dwelling on certain objects, that habit may continue when the immediate cause is removed. Under such circumstances, by continual and unwearied attention of those about the patient, it may surely be possible to induce a different train of images. To suppose that this can be effected by logical reasoning, would be giving the madman, or the convalescent madman, an advantage over the sound mind; for who would endeavour, by mere logic, to convince a disconsolate widow, that having suffered only the common lot of humanity, her distress will never restore her husband; and that the only remedy being the supplying of his place, it would better become her to regard her health, that she might appear more advantageously to the men. But if she should gradually be brought into the company of other widows, and observe the cheerfulness which they have have acquired by time, she may see, without being told it, that widowhood is a distress, which, like all others, may be relieved by time. Thus, if a man has become mad by the loss of title, consequence, or wealth, and the first shock has been sufficient to injure the structure of the brain, it will be in vain to propose any other remedies than such as are directed to the immediate relief of that organ. When we have reason to suppose we have so far succeeded, if we find the mind still dwells upon stars and garters, or the chairing at elections, who would be so foolish as to tell him gravely, that probably the ribbon may only cover an aching heart. All this and much more, is well known, has been often repeated, and may be admitted by the madman at the very time the event happened which produced his disease. The object should therefore be, as Mr. Haslam remarks, to divert the mind from this train of ideas; not, we would add, by keeping every thing at a distance which would revive them, for that is impossible, as the patient would be always calling them up; but by rendering him familiar with them, and in such a way that they may lose all their terrors. And this can only be done by an accurate knowledge of the patient's turn of mind, and by an address, the acquisition of which must be the effect of constant study. We shall conclude this part of our subject by one general remark. It is universally admitted, that mad people recover best, and are most manageable, when associated together. To what are we to impute this but to a consciousness, not impressed by argument but their senses, that madness is by no means an uncommon complaint; that there are many whose situation is worse than their own; and that most of those they see are in a convalescent state: that no injury is offered to them, and that they are prevented from injuring others. Thus they lose insensibly many of the terrors of their situation, and by degrees feel something like hope of a restitution of their liberty and self management.

On the probable event of the disease, Mr. Haslam, with much propriety, confines himself to written documents. It appears generally, that the more advanced the age of the patient, the less probability there is of recovery. The same happens in most other diseases. The more common periods of attack is from 40 to 50 years of age. The climacteric changes have considerable influence in the occurrence and cessation of the disease. The acute form promises a more certain recovery than the chronic.

On the subject of religious melancholy, our Author's prognostic is particularly gloomy; indeed, this is a necessary inference from the disease and the cause. The common affairs of life, however perplexing, may be reconciled by us; they appear in a *tangible form*; we can familiarize ourselves to them, and learn with some certainty the worst. But the terrors of a future life must be ideal; nor can we prevent their occurrence with all the horrors in which the state of the patient will dress them.

Of the management and remedies we shall only say, that whatever our Author proposes, appears perfectly rational, and, we doubt not, has been by himself reduced to practice.

---

*Reports on the Effects of a peculiar Regimen on Scirrhous Tumours and Cancerous Ulcers.* By WILLIAM LAMBE, M. D. Fellow of the Royal College of Physicians. 8vo. pp. 190.

A SHORT Preface precedes these Reports, by which we learn that the Author considers them as a Supplement to his former work. In this preface, it is with much concern that we find the former labours of Dr. Lambe have been treated with undue severity.—When a writer relates the result of his inquiries with candour, he is entitled not only to fair treatment but to the thanks of the public. Dr. Lambe may in a particular manner claim this, as he has been minute in detailing his facts, whether the result has been successful or not. We have already expressed our opinion of the Author's former publication; of his good intentions there can be no doubt, any more than of the faithfulness of his narrative. We must add, there cannot be a doubt of the importance of his relations; for though they have not been sufficient to convince us of the advantages he expects to derive from his peculiar regimen, yet in this, as in all other diseases, a faithful report of their progress is often the most valuable present a medical writer can offer to his brethren.

But whilst we make these concessions to our Author, he must not be offended if we differ from him in his opinions, and even ascribe his success to causes of which he does not seem aware. At the same time we ought to confess, that we are not without our own favourite theories, which to others may appear whimsical; and if any thing will authorize a bolder view of an intricate subject, it is surely the reflexions on this most dreary and painful disease.

The present work begins with an inquiry, whether it is natural for men to drink? This is certainly an improvement on the tendency of the former work; for if common water is deleterious, it seems impossible to say how it is to be avoided but by not drinking any thing. As, however, Dr. L. has left this a subject in some measure of conjecture, we shall not dwell upon it at present, especially as he conceives any attention to what we drink, or to not drinking at all, would be insufficient without another article of discipline, to his former ignorance of which he imputes his want of more general and permanent success. After acknowledging that epilepsy has continued with unabated force during a course of distilled water; that gouty paroxysms had recurred, though with abated violence and at longer intervals; that consumptive symp toms, painful affections of the head, and even mania, have mad their first appearance in subjects, whose faithful adherence to distilled water he had no reason to doubt,—he remarks,

" But

"But these facts, however discouraging, could by no means subvert and nullify the very obvious and evident advantages derived from the use of pure liquids: above all, it could not overturn that very striking and remarkable fact, that a cancerous ulcer, which had been spreading for five months, became immediately stationary upon using distilled water; and, as it subsequently appeared, continued so till the last moment of life. In this observation, which has now been repeated so often, that I doubt not, but that, with certain limitations of age, it will prove uniformly true, it was impossible to attribute any thing to fancy, or to that species of self-deception that men are apt to indulge in, who are attached to a favorite hypothesis. It seemed clearly to follow from it, that the perpetual and progressive increase, which so strongly marks this cruel disease, is to be ascribed to the perpetual and never ceasing activity of the fluid matter, which we are hourly taking in.

"It seemed probable then, that the symptoms which were unaffected by this course should be attributed to one of two other causes, or to both of them united. First of all, other matters, equally injurious to the system, might also be in action; or, secondly, the disorganization of the whole system might be so great, that its restorative powers might be totally destroyed. And such, I fear, will be found the truth, wherever there is great lesion of the internal parts of the system.

"Now one order of symptoms having been so clearly traced to the operation of the liquid *ingesta*, it became the most obvious suspicion, that errors in the nature and qualities of the solid aliment might be the occasion of some of the other very severe and intractable symptoms.

"Vegetable matter forms the nutriment of the great bulk of the human race; at the same time, all men, whose circumstances permit them to indulge their appetites, use no small proportion of the flesh of animals, as an article of diet. Man has, in consequence, been deemed to hold a middle rank between carnivorous and herbivorous animals; and physicians have been very ready to suppose that individuals possessed constitutions essentially different; some requiring an increased proportion of vegetable food, or even a total abstinence from flesh; others, a treatment entirely opposite. Nor have such opinions been adopted upon slight foundations. The simple abstinence from flesh has been known sometimes radically to cure, more frequently to palliate, some of the worst diseases to which our frame is subject, as epilepsy, gout, rheumatism, hypocondriasis, pulmonary consumption, and, in general, all disorders, which are accompanied with signs of turgescence, or with a dry and feverish habit. But in diseases accompanied with little or no fever, where there are signs of debility and diminished action, a more plentiful diet of animal food has often been attended with effects apparently the most happy. The attempts to adopt a vegetable regimen has often produced so much lowness and

 debility debility, amounting even to fainting, and other alarming symptoms, as to excite an almost unconquerable persuasion, that there are certain constitutions, to which animal food is indispensible, and with which a vegetable regimen is absolutely incompatible.

"But notwithstanding these apparent varieties of the human constitution, I am perfectly convinced, that those philosophers, (few in number, but powerful in name and in argument) are in the right, who have maintained, that man is in his proper nature strictly to be ranked among the herbivorous animals; and that the use of the flesh of animals is a deviation from the laws of his nature, and is universally a cause of disease and premature death. The almost universal appetite which is shewn for flesh, approaching nearly to the strength of instinct, (the fact which is the strongest argument in its favour,) is formed partly by indulgence; but it is produced, I believe, much more by the natural instincts and propensities of man being destroyed or perverted by his situation and his habits. It is certain that other animals can be nourished by matter which is quite foreign to their nature and original propensities, and by habit they contract a relish and attachment to their new diet. Carnivorous animals, (the dog, for example) has been fed, and apparently well nourished, upon vegetables; herbivorous animals (as the horse, the cow, the sheep, or the ape) have been fed upon flesh, and even upon fish. But whether animals thus forced out of their natural habits, are as healthy, and live as long as when following the guidance of instinct, has not, I believe, been ascertained, or much thought about. One striking example, however, of its pernicious consequence is continually before our eyes. The hog is naturally herbivorous; he is formed to turn up the earth, and to collect his nourishment from roots and fruits. Under the care of man he has become omnivorous; but at the same time, he is become the most unhealthy of all our domestic animals.

"Take the full grown man, changed and transformed as he is, by a variety of causes, and we should infallibly conclude that the desire of animal food is one of his most powerful instincts. But in children it is far otherwise. They are mostly indifferent to animal food; it often affects them with extreme disgust: milk, fruits, and vegetables are their delight. This of itself is enough to infuse a strong suspicion, that the appetite and relish for animal food is an artificial appetite, produced by the gradual operation of foreign causes.

"It is allowed, that by a confinement to vegetable food, the senses of sight, smell, and touch, become more acute; that the intellectual faculties are more clear; the skin more perspirable; the body more active; if such a regimen does not give an exemption from acute diseases and premature death, it upon the whole is favourable to longevity. It is certain also that it conduces to form a milder character, dispositions more benevolent, and morals more pure. If then animal food makes the senses less acute, acute, the intellect less clear; if it produces a dry skin and a lethargic habit; if it renders the animal short-lived, and, whilst he lives, depraves his character; it would seem to require no further proof, that it is a species of aliment not suited to the proper nature of man. Pathological facts are still stronger. If epilepsies, for example, have been cured by abstaining from animal food; if paroxyms of gout have been subdued; in these examples at least, the use of this aliment was obviously the cause of these most dangerous and painful affections. The question then recurs with increased force,—Is it possible that the direct and immediate cause of such great and obvious mischiefs can be innocent and useful in any case whatever?"

The Author afterwards proceeds to prove, by what is known of comparative anatomy and physiology, that man was intended to be only herbivorous.

"Anatomy, it is said, demonstrates that man was intended by his Maker to use a mixed diet. Great authorities are quoted in behalf of this position. "The structure of man"—says the illustrious Haller (in whose defence it may be said, that he lived at a time when the study of comparative anatomy was in its infancy) the "structure of man is placed between that of carnivorous and herbivorous animals, and partakes of both, approaching nearer, however, to that of the herbivorous." In like manner, Mr. Home (for whom the same apology cannot be made) has said, that the human stomach is the link between those of animals, which are fitted to digest vegetable substances, and those that are entirely carnivorous: but he acknowledges, not very consistently, it would seem, that its internal structure exactly resembles that of the monkey and squirrel, two animals which live entirely on vegetable matter.

"Facts, however, seem in direct opposition to this hypothesis. More than a century ago, Tyson and Cowper had observed the exact resemblance both external and internal between man and the species of ape, called orang-outang. This resemblance is so close, that Buffon, after noticing some points of difference, observes, "all the other parts of the body, the head and limbs, are so perfectly similar to that of man, that we cannot compare them without admiration, nor without being astonished, that from a conformation so similar, and an organization which is absolutely the same, there should not result the same effects. Man and the orang-outang are the only animals, in which the brain, the heart, the lungs, the liver, the spleen, the pancreas, the stomach, the bowels, are absolutely alike: the only ones which have a vermicular appendix to the cæcum; in fine, the orang-outang bears a closer resemblance to man, than to any other animal, even than to baboons and monkeys.

"Hear the sentiments of the experienced M. Daubenton, the associate of Buffon, and the first writer who rendered the study of anatomy subservient to natural history.

"The example of carnivorous animals is not conclusive to determine the nature of the aliments most proper for man, because, from the conformation of these animals they have fewer relations than those animals who live on vegetables. I have clearly ascertained this fact by comparative anatomy, in the dissection of a great number of animals of different species.'

"Apes are the animals who differ the least from us in the general conformation of their bodies, particularly in that of the mouth, of the teeth, of the tongue, of the throat, of the œsophagus, of the stomach, and of the intestinal canal. This analogy, which I have carefully traced between man and the ape species, doubtless takes place in the functions of digestion, as well as in the structure of the alimentary canal; consequently there is every reason to infer a similar analogy in the nature of their respective food. But the wild apes, who range at liberty in their native woods, live solely on vegetable productions; it is then highly probable that man, in a state of pure nature, living in a confined society, and in a genial climate, where the earth required but little culture to produce its fruits, did subsist upon these without seeking to prey on animals.

"To examine the question a little more closely.—"These two classes," (the carnivorous and herbivorous) says Haller, "are said to differ in the stomach, teeth, intestines, and principally in the form of the *intestinum cæcum*." To shorten the discussion, I will confine it within these limits; though they are much too narrow. A complete view of the subject ought to survey man in the whole of his frame, in his more important relations, in the structure of his mind, and the genuine unsophisticated emotions of his heart."

A comparative view is next offered of the stomach, the teeth, and the intestines of man and other animals, by which it appears that man only resembles those animals which are truly herbivorous.—It would seem as if our Author were of opinion that man, though the inhabitant of every part of the globe, is fitted only for the warmer climates, where he may enjoy a perpetuation of that food which is only suited to his health and longevity, and in which he is not only less desirous of animal food, but more immediately feels the ill effects of indulging in it.

"But, continues our author, a second cause, which is common to all climates, and which will be found to be still more powerful, is the use of watery liquids, as a substitute for the fruits and vegetable juices, with which man would, I believe, in a state of primæval simplicity, at once satisfy the appetite of hunger, and prevent thirst. The poison thus introduced into his body directly deranges the sensorium, alters his feelings, and gives a new and unnatural direction to all his propensities. It produces a great change on the powers of digestion; and with this it effects a corresponding change in the desires and aversions. Vegetable matter, which, to the stomach of a healthy child, is the most delightful, the most nutritive and strengthening aliment, gradually seems to lose its power; it ceases to impart either strength or pleasure. In a state of manhood, to many it is an object of disgust, to almost all of indifference, indifference. It excites flatulence, and often gives pain and uneasiness; and the power of digesting it becomes more and more destroyed. To render it tolerable, it must be heated and macerated: by these means it is made more soluble, and digestible with greater speed. But by these same means its sweet and nutritious juices are either decomposed or extracted; and weighty reasons may, I think, be given, to shew that, in this condition, it neither imparts the strength nor the nourishment that it would do, when used, as it is by the animals, without any preparation. How astonishing is this revolution! How inconceivable, that the only species of food, which, previous to the invention of arts, it was in the power of a human being to obtain;—that the only species of food, on which the primæval race subsisted, during the silent lapse of ages;—that the species of food, which we know affords a healthy nourishment at this present day to many races of men,—how inconceivable is it, that in all civilized and crowded communities it is not merely disregarded, but seems to become truly indigestible, and on many to assume the force and activity of a true poison!

"Now, that this is truly the effect and consequence of using water in its ordinary condition, is not an imaginary hypothesis, but a serious truth, the result of careful and repeated experience. It will be found experimentally true, that by using distilled water, the power of digesting vegetable matter will be restored and improved; that the stomach will gradually be enabled to digest it, even raw, and without any condiment or other preparation; that with the power of digestion, the inclination to vegetable food will be renewed; that it will be easy under such a system entirely to subdue the desire and craving for animal food; that, finally, what was at first looked upon with antipathy and disgust, will, by habit, be rendered most easy and most delightful.

"If the sketch, I have drawn, of the great revolution of habits, which the human species has undergone be just, it gives at once a complete solution of the corresponding change, which has been introduced into the moral and physical constitution of man. If he can be restored to his primæval integrity, he must carefully retrace his steps, and regain the path, from which he has so widely deviated. In enjoining him then to confine himself to vegetable food; to use it, as much as his feelings of comfort will permit, in the form presented him by the parental hand of nature; and to avoid the use of all fluid matter contaminated and loaded with putrefaction; I am but bringing him back, as nearly as is possible in a complicated state of society, to the condition which he has quitted; or, to speak more truly, I am leaving him in the possession of all that is valuable in social and polished life, and presenting to him the consoling prospect of being rescued from the load of innumerable evils, which it has entailed upon him.

"But it cannot be expected, that any thing but facts, the most precise and authentic, can make men apprehend the possibility of effecting so great a change in the human constitution. So much are are we the slaves of habit, that every thing new is ridiculous. But in truth, the theory I have chalked out, is founded upon facts, all of them of unquestionable authenticity, and most of them of perfect notoriety. No facts can be more authentic than the utility of the pure natural springs, such as those of Malvern, in a variety of obstinate and intracticable disorders. Scrophulous tumours, and ulcerations, and other defedations of the skin, have afforded the most obvious proofs of their powers; and if these facts are not universally known, it is obviously owing to two causes. 1st. Such springs being rare, their use is necessarily very limited, and their reputation local: 2d. All regimen by water alone is essentially defective; and, in consequence, the benefit will be partial and imperfect; and there will be many examples of failure. Still, the real power being unquestionable, to attempt to imitate these waters, or rather to improve upon them, and to determine experimentally whether their utility is the effect simply of their purity, must be deemed the first step towards the establishment of a regular system of dietetics. No facts again can be more authentic or more notorious, than the utility of a vegetable regimen in other diseases, some of them of the most severe, painful, and dangerous of any that the human frame can undergo. But on ulcerations, in particular, this regimen has had little or no beneficial influence: insomuch that, in scrophulous complaints, it has been deemed (upon no just foundation, I am persuaded) to be directly injurious.

"It seems therefore the most simple deduction of reason and common sense, to expect, from the combination of these two systems, effects, which are not to be hoped for from either of them singly, and which it is in vain to look for in the operations of drugs."

We have given these comparatively few arguments and illustrations, as far as our limits would admit, in the Author's own words; and we would gladly extend them further, as they are written with much perspicuity and candour. But it is more to the purpose that we should produce those cases in which advantage has been derived from the use of the proposed regimen.

The first case has in part appeared in the work to which this is a supplement; we shall transcribe it in as few words as is consistent with accuracy and justice to the author. The patient was married, but never had children. At the age of 44 she first perceived a hardness in her right breast; it became painful, and her health and appearance declined considerably. In the latter end of June, 1804, not less than a twelvemonth from the time the disease was first discovered, she began the use of distilled water. After a month's trial the superficial ulcers formed from small tumours immediately under the skin, and unconnected with the scirrhus, became stationary. In October following, Dr. Lambe saw her. —

"At this time the schirrosity occupied the whole of the glandular substance of the breast, which was of a stony hardness, with an irregular tuberculated surface; but it was not of more than the natural size, nor had it formed any adhesions to the side. The skin skin over the schirrous tumour was entire, and of its natural colour: it was adherent to the tumour beneath. The surrounding little tumours had a thickened and elevated margin, and at their central parts the cutis was detached from the base; consequently, at this part they formed a small, and apparently, an empty vesicle. That which had now been ulcerated some months, was still of the same size; its base was clean, but its edges thick and callous; a serous fluid exuded from it, but not very copiously: it gave little or no uneasiness. The smaller ulcer was still no larger than a pea. The axillary glands were swollen and hard. She sometimes suffered very severe and oppressive pain in the diseased breast, which would last a great many hours successively. She had been so afflicted for two days previous to my visit; but at that time the paroxysm had subsided.

"The constitutional symptoms were still more unfavourable than the local. Her strength was so much reduced, that she was confined to her room. Her habit was naturally spare, and she was now much emaciated. The gums were loose and spongy, and she had lost several of her teeth. The complexion was pallid; and the pulse quick and feeble. She had suffered much from pains of the limbs, resembling rheumatism, in consequence of which, the lower limbs, in particular, were much enfeebled; nor had she a due degree of strength in the hands and arms. The most favourable sign about her was, that the appetite was but little impaired."

She had then continued her course more than three months, and the effect had been very satisfactory. But notwithstanding these flattering appearances, in the following month, pains, sickness and vomitting occurred every sixth day, and her friends were apprehensive of an immediate dissolution. From that time the prospect mended. In April 1805, her health improved so much that she undertook a journey, with the perverse intention, notwithstanding her amendment, of putting herself under the care of the late Dr. Rowley, whom she had occasionally consulted by letter. She remained about a month under Dr. R's care, during which, and for two weeks more, she discontinued her regimen.— For three or four weeks she found no inconvenience; afterwards, the change caused a sense of general uneasiness and irritation, the strength sunk, and she became subject to constant perspiration. Under these circumstances, she resumed her distilled water, and placed herself under Dr. Lambe's care. "At the latter end of May, 1805, I found the local disease, says our author, much improved. All pain in the schirrous tumour and in the axillary glands had ceased. The thickened edge of the ulcer, which had begun to yield ten or twelve weeks before, was entirely removed, so that the sore presented a flat, superficial, granulating surface; but the granulations were pale and feeble. I could not perceive that the ulcer had at all spread from the time that I had before seen it. The condition of the little ulcer was precisely similar, being still no larger than a pea. No more of the little cancerous tumours mours of the skin had ulcerated; but several more of them had appeared, of which, however, the summits had no appearance of vesication. There had been, I was told, a considerable tumefaction of the skin, somewhere between the breast and the shoulder; but this was entirely removed.

"But though the health had improved considerably, it was far from having acquired stability. The legs were slightly œdematous; there was a general paleness; the bowels were deranged; and the debility was considerable. The arms, in particular, were so weak, that she could not put them behind her head, to adjust her dress. The liver was much tumefied; and there were some grounds to suspect that water was beginning to be effused into the cavity of the abdomen."

The principal ulcer discharged matter varying from pus to serum; the skin frequently formed over the sore, and as frequently came away. All this time the patient's health declined. She suffered severe rheumatic pains, from which she had occasional remissions. In the following August it became evident that water was collected in the abdomen. These symptoms subsided in the succeeding month; but about the same time her return of sickness was attended with apthæ in the mouth, tongue, and fauces, which seemed to extend to the œsophagus, stomach, and intestines. In the following month (October) she was so much recovered, even from these unfavourable symptoms, that she returned into the country, where she gained strength enough to dine below, go into the garden, and enjoy the society of her friends. The ulcer mended, and her general appearance was favorable; but some uneasiness and tumefaction about the abdomen, gave grounds for apprehending that this respite was fallacious; and about Christmas she had another attack, from which she never recovered. Though not in much pain, her appetite was lost, and what she took into her stomach was rejected. Water accumulated in the abdomen, though it was free from tension, and her urine and perspiration profuse. In this state she languished till the beginning of February, when she died.

The Author's remarks on this case, we must admit, are candid, though the conclusion is much too decided for us. After admitting that the termination of the case proves there was something radically defective in the regimen, he urges, that as during the space of eighteen months the tumour neither increased nor formed adhesions, nor ulcerated; as the ulcer which had spread for five months remained stationary for the subsequent eighteen, lost its carcinomatous character, and assumed the appearance of a healing sore; as the skin, where the disease begun, became entirely sound, it seems to follow irresistably, that these the proper symptoms of cancer are occasioned by the use of common water, and are suspended by the use of distilled water: and "that therefore *common water is the vehicle in which the poison of cancer is introduced into the system.*"

( To be continued. )

